# Immune checkpoint inhibitor-associated celiac disease

**DOI:** 10.1136/jitc-2020-000958

**Published:** 2020-06-24

**Authors:** Yousef R Badran, Angela Shih, Donna Leet, Meghan J Mooradian, Alexandra Coromilas, Jonathan Chen, Marina Kem, Hui Zheng, Jennifer Borowsky, Joseph Misdraji, Mari Mino-Kenudson, Michael Dougan

**Affiliations:** 1 Department of Medicine, Massachusetts General Hospital, Boston, Massachusetts, USA; 2 Harvard Medical School, Boston, MA, USA; 3 Department of Pathology, Massachusetts General Hospital, Boston, Massachusetts, USA; 4 Division of Oncology, Department of Medicine, Massachusetts General Hospital, Boston, Massachusetts, USA; 5 Department of Medicine, Columbia University, New York, New York, USA; 6 Biostatistics Center, Massachusetts General Hospital, Boston, MA, USA; 7 Division of Gastroenterology, Department of Medicine, Massachusetts General Hospital, Boston, Massachusetts, USA

**Keywords:** costimulatory and inhibitory T-cell receptors, immunotherapy, inflammation

## Abstract

**Background:**

Rare cases of immune checkpoint inhibitor (ICI)-associated celiac disease (ICI-CeD) have been reported, suggesting that disruption of tolerance mechanisms by ICIs can unmask celiac disease (CeD). This study aims to characterize the clinicopathological and immunophenotypic features of ICI-CeD in comparison to ICI-associated duodenitis (ICI-Duo) and usual CeD.

**Methods:**

A medical and pathological records search between 2015 and 2019 identified eight cases of ICI-CeD, confirmed by tTG-IgA. Nine cases of ICI-Duo, 28 cases of moderate CeD, as well as 5 normal controls were used as comparison groups. Clinical information was collected from the electronic medical records. Immunohistochemistry for CD3, CD8, T-cell receptor gamma/delta (γδ), programmed death ligand 1 (PD-L1), and programmed death 1 (PD-1) were performed, with quantification of intraepithelial lymphocyte (IEL) subsets in three well-oriented villi. CD68, PD-L1, and PD-1 were assessed as a percentage of lamina propria surface area infiltrated by positive cells. Statistical significance was calculated by the Student’s t-test and Fisher’s exact test.

**Results:**

The eight patients with ICI-CeD (F:M=1:3) and nine patients with ICI-Duo (F:M=5:4) presented similarly with diarrhea (13/17) and abdominal pain (11/17) after a median of 1.6 months on ICI therapy. In patients with ICI-CeD, tTG-IgA ranged from 104 to >300 IU/mL. Histological findings in ICI-CeD and ICI-Duo were similar and included expansion of the lamina propria, active neutrophilic duodenitis, variably increased IELs, and villous blunting. Immunohistochemistry showed that the average number of IELs per 100 enterocytes is comparable between ICI-CeD and ICI-Duo, with increased CD3^+^ CD8^+^ T cells compared with normal duodenum but decreased γδ T cells compared with CeD. Average PD-L1 percentage was 9% in ICI-CeD and 18% in ICI-Duo, in comparison to <1% in CeD and normal duodenum; average PD-1 percentage was very low to absent in all cases (<3%). On follow-up, five patients with ICI-CeD improved on a gluten-free diet (GFD) as the sole therapeutic intervention (with down-trending tTG-IgA) while the other three required immunosuppression. All patients who developed ICI-Duo received immunosuppression with variable improvement in symptoms.

**Conclusions:**

ICI-CeD resembles ICI-Duo clinically and histologically but shares the serological features and response to gluten withdrawal with classic CeD. Immunophenotyping of IELs in ICI-CeD and ICI-Duo also shows similar CD3, CD8, γδ T cell subsets, and PD-L1 populations, all of which differed quantitatively from usual CeD. We conclude that ICI-CeD is biologically similar to ICI-Duo and is likely a variant of ICI-Duo, but treatment strategies differ, with ICI-CeD often improving with GFD alone, whereas ICI-Duo requires systemic immunosuppression.

## Background

Celiac disease (CeD) is a systemic autoimmune disease characterized by small intestinal enteropathy precipitated and propagated by dietary gluten in genetically susceptible individuals.^1–3^ When immunogenic gluten peptides traverse the intestinal lumen, they can elicit both an innate and an adaptive immune response, leading to the clinical and histological manifestations of CeD.^4^ Patients often present with a constellation of intestinal and/or extraintestinal manifestations, although some may be asymptomatic at diagnosis, and histological confirmation by the presence of intraepithelial lymphocytes (IELs) and villous atrophy is often necessary.^3 5^ Measurement of serum IgA antibodies to tissue transglutaminase (tTG-IgA; or IgG class in patients with IgA deficiency) is the first recommended screening test for patients with suspected CeD.^1 2^

The immune regulatory proteins cytotoxic T lymphocyte antigen (CTLA)-4 as well as programmed death (PD)−1 and its ligand PD-L1 are important immune regulatory proteins collectively referred to as immune checkpoints receptors. Studies have shown that these pathways can be appropriated by malignant tumors as a mechanism to circumvent antitumoral immune responses.^6^ Underscoring the importance of these pathways, immune checkpoint inhibitors (ICIs) have demonstrated impressive clinical activity, with monoclonal antibodies targeting CTLA-4, PD-1, and PD-L1 now approved for the treatment of diverse cancers.^7 8^

Although ICIs demonstrate remarkable efficacy against advanced cancers by enhancing antitumor effector functions of T cells,^9^ inhibition of these regulatory receptors leads to loss of tolerance and a wide spectrum of inflammatory toxicities known as immune-related adverse events (irAEs). When severe, irAEs can necessitate ICI therapy interruption, discontinuation, and treatment escalation with powerful immunosuppressive agents.^10–13^ Isolated ICI-associated duodenitis (ICI-Duo) has been reported after treatment with different immunotherapies,^14 15^ often with non-specific histological features.^14–16^ Interestingly, rare cases of patients with de novo elevation of serum tTG antibodies in the setting of treatment with ICIs have been reported.^17–19^

The nature of the relationship between serum tTG antibodies and ICI-Duo is currently unclear, but it has been hypothesized to reflect treatment-emergent CeD.^17 20^ Whether ICI-associated celiac disease (ICI-CeD) reflects new onset gluten sensitivity or is the first manifestation of previously asymptomatic CeD remains an open question. As the clinical and histological manifestations of gastrointestinal (GI) irAE are broad and may mimic other primary diseases (including CeD), this study aims to elucidate the clinicopathological and immunophenotypic characterizations of patients with ICI-CeD in comparison to ICI-Duo and conventional CeD.

## Patients and methods

### Patients

A comprehensive search of the clinical and pathology records at the Massachusetts General Hospital (MGH) between the years 2015 and 2019 identified eight patients with ICI-CeD (confirmed by serological testing for tTG-IgA), along with nine patients with ICI-Duo defined as pathologically confirmed duodenal inflammation while on ICI therapy per routine standard of care or clinical trial protocol. Patients with concurrent colitis were excluded from the ICI-Duo cohort. All patients had been referred to the gastroenterology service for new GI problems after receiving ICIs. Positive infectious disease testing was an exclusion criteria, and included *Clostridium difficile* testing by toxin A/B immunoassay, stool ova and parasites examination, stool culture, and serum cytomegalovirus viral titers. This search was inclusive of a well-defined cohort of 376 patients with melanoma treated with ICI at the MGH Cancer Center between 2013 and 2017, which was used to calculate approximate frequencies of specific luminal toxicities (Melanoma Cohort). Additionally, a search of the pathology records identified age-matched, otherwise healthy patient controls with typical CeD (modified Marsh 3b) as well as non-CeD controls with normal duodenum.

### ICI-CeD and ICI-Duo definitions

ICI-CeD was defined as clinical evidence of duodenitis with tTG antibody positivity that developed after ICI administration, with histopathological confirmation when available. ICI-Duo was defined as clinical and histological evidence of duodenitis with a negative tTG antibody. Patients with duodenitis who did not have tTG antibody measured and were treated with standard management for immune-related enterocolitis with appropriate response were included in the ICI-Duo group. Active colitis was ruled out in patients presenting with diarrhea through lower GI endoscopic evaluation.

### Data collection

Details of the medical and oncological histories were reviewed in the electronic medical record. Data pertaining to ICI-Duo and ICI-CeD development and management include: presenting symptoms, laboratory workup, corticosteroid dose and number of steroid taper attempts, and infliximab use. Laboratory parameters including transferrin saturation, vitamin D, vitamin B_12_, and transaminases were captured between 2 weeks prior and 1 year after diagnosis of GI toxicity. Antitumor outcomes including overall survival (OS) and progression-free survival (PFS) were calculated and reviewed by a medical oncologist (MJM).

### Histology and immunohistochemistry

Duodenal biopsies were reviewed by two GI pathologists (AS and MM-K) and assessed for villous blunting, neutrophilic duodenitis, expansion of the lamina propria, intraepithelial lymphocytosis, and surface erosion or ulceration. An immunohistochemical panel was performed on a representative slide of duodenum for each selected case and control case, consisting of the following markers and staining conditions: CD3 (Leica; RTU; ER2, 15 min), CD8 (Leica; RTU; ER1, 20 min), T-cell receptor δ (Santa Cruz Biotech; 1:200; ER2, 40 min), PD-L1 (Cell Signaling; 1:200; ER2, 20 min), PD-1 (Cell Marque; 1:200; ER2, 30 min), and CD68 (Biocare; RTU; ER1, 20 min).

Quantification of the IELs is reported as an average number of CD3, CD8, and δγ positive cells per 100 enterocytes in three well-oriented villi. Quantification of lamina propria lymphocytes is reported as an average number of CD3 and CD8 cells per high power field (HPF; 400×) in three HPFs. Assessment of CD68, PD-L1, and PD-1 is reported as a percentage of lamina propria surface area infiltrated by positive cells in the biopsy.

### Statistical analysis

Patients were grouped in two primary groups for analysis: ICI-Duo and ICI-CeD. Statistical analysis, including Fisher’s exact test, analysis of variance, and Student’s t-test, was performed using GraphPad Prism V.8 (GraphPad Software, La Jolla, California, USA) or Python SciPy V.1.4.1. Data are expressed as “mean±SD,” “mean±SE,” or “median (range)” where appropriate.

## Results

### Demographics and baseline characteristics

We identified nine patients who developed ICI-Duo (median age at presentation: 60 years; F:M=5:4) and eight patients who developed ICI-CeD (median age at presentation: 55 years; F:M=1:3; table 1). The majority (71%) of patients received ICI for treatment of metastatic melanoma. In the entire cohort, eight patients received single-agent PD-(L)1 inhibition, five received single-agent CTLA-4 inhibition, and four received combination anti-PD-(L)1/CTLA-4 inhibition. Prior to developing GI symptoms on the current ICI regimen, only one patient had received a different ICI regimen in the ICI-CeD group (n=1/8), compared with five patients in the ICI-Duo group (n=5/9). Among the patients who developed ICI-CeD, two had non-small cell lung cancer, one had extraskeletal myxoid chondrosarcoma and one had tonsillar squamous cell carcinoma. No patients had known metastasis to the GI tract on initiation of immunotherapy (table 1).

**Table 1:** Demographics and baseline characteristics

Characteristics	ICI-duodenitis (n=9)	ICI-celiac disease (n=8)	P value
Median age at presentation, years (range)	60 (29–71)	55 (44–73)	0.804
Sex (F:M)	5:4	2:6	0.334
Malignancy type, N (%)			
Melanoma	8/9 (89)	4/8 (50)	0.131
Lung	0/9 (0)	2/8 (25)	0.205
Other	1/9 (11)	2/8 (25)	0.576
Stage at initiation of ICI			
III	3/9 (33)	3/8 (37.5)	>0.999
IV	6/9 (67)	5/8 (62.5)	
Metastatic sites at ICI initiation			
Lung	3/9 (33)	3/8 (37.5)	>0.999
Liver	2/9 (22)	2/8 (25)	
Brain	2/9 (22)	1/8 (12.5)	
Other	4/9 (44)	0/8 (0)	
None	3/9 (33)	3/8 (37.5)	
History of prior immunotherapy use	5/9 (56)	1/8 (12.5)	0.132
Immunotherapy at time of symptom onset, N (%)			
α-CTLA-4	4/9 (44)	1/8 (12.5)	0.294
α-PD-(L)1	3/9 (33)	5/8 (62.5)	0.346
Combined therapy	2/9 (22)	2/8 (25)	0.999
Autoimmune disease history, N (%)	0/9 (0)	0/8 (0)	>0.999
Luminal GI disease history			
GERD	4/9 (44)	3/8 (37.5)	>0.999
H.pylori PUD	0/9 (0)	0/8 (0)	>0.999
IBD	0/9 (0)	0/8 (0)	>0.999
Celiac disease	0/9 (0)	1/8 (12.5)	0.47
Family history of CeD	0/9 (0)	2/8 (25)	0.205

The p value was calculated by Student’s t-test and analysis of variance method for numerical covariates and Fisher’s exact for categorical covariates where appropriate. Other malignancy types for immune checkpoint inhibitor-associated duodenitis (ICI-Duo): Hodgkin lymphoma (n=1). Other malignancy types for ICI-CeD: extraskeletal myxoid chondrosarcoma (n=1) and tonsillar squamous cell carcinoma (n=1). Patients with (none) listed for metastatic sites at therapy initiation had stage III disease. Other metastatic sites for ICI-Duo: adrenal gland (n=1), bone (n=2), and peritoneum (n=1). History of prior immunotherapy use was identified as any ICI used prior to the current regimen. Combined therapy denotes that patients recieved ipilimumab and a programmed cell death receptor (ligand)-1 (PD-(L)1) inhibitor as standard of care or on an investigational protocol. Family history of celiac disease denotes CeD in first or second degree relative.

CTLA-4, cytotoxic T cell associated antigen 4; GERD, gastroesophageal reflux disease; GI, gastrointestinal; H.pylori, *Helicobacter pylori*; IBD, inflammatory bowel disease; PUD, peptic ulcer disease.

The two groups had comparable histories of extraintestinal autoimmune disease and luminal GI disease, and no patients had a history of liver disease. One patient who developed ICI-CeD was known to have clinically asymptomatic CeD that flared after ICI therapy. Of the eight patients who developed ICI-CeD, two had a family history of CeD in a first degree relative.

To determine an approximate frequency of ICI-CeD and ICI-Duo, the Melanoma Cohort was used as a defined population. Of the 376 patients in this cohort, 123 patients were sent to endoscopy and 96 of these patients had symptoms of possible ICI toxicity. Most patients with suspected toxicity were found to have mucosal inflammation on biopsy (63, 65.6% of total). Of these 63 patients with inflammation, 49 had colitis or enterocolitis (77.8%), 8 had enteritis or gastroenteritis (12.7%), 3 had isolated gastritis (4.8%), 1 had esophagitis (1.6%), 1 patient had new onset CeD (1.6%), and 1 patient had unclassified inflammation (1.6%). The patient with ICI-CeD represented 0.3% of the total Melanoma Cohort exposed to ICIs.

### Clinical presentation

The most common presenting symptoms for both ICI-CeD and ICI-Duo were diarrhea and abdominal pain. The median time to symptom onset after ICI initiation was 48 days (range 20–409 days) in patients with ICI-Duo, compared with 82.5 days (range 18–679 days) in patients with ICI-CeD. Monotherapy with PD-(L)1 blockade led to a later onset of symptoms (median 159.5 days, range 19–679 days) compared with monotherapy with CTLA-4 or combined therapy with CTLA-4 and PD-(L)1 blockade (median 35 days, range 18–144 days; table 2). Extraintestinal manifestations including vitamin deficiencies, dermatitis herpitiformis, transaminase elevations, and constitutional symptoms, which were present between 2 weeks prior to diagnosis to 1 year after diagnosis, are reported in table 2.

**Table 2:** Immune checkpoint inhibitor-associated duodenitis (ICI-Duo) and ICI-associated celiac disease (ICI-CeD) clinical course

Characteristics	ICI-Duo (n=9)	ICI-CeD (n=8)	P value
Time to symptoms onset (median, days)			
α-CTLA-4	41.5 (n=4)	31.0 (n=1)	0.616
α-PD-(L)1	221 (n=3)	119 (n=5)	0.79
Combined therapy	27.5 (n=2)	81 (n=2)	0.487
Overall	48	82.5	0.623
Symptoms at diagnosis			
Abdominal pain	6/9 (67%)	5/8 (62.5%)	>0.999
Diarrhea	7/9 (78%)	6/8 (75%)	>0.999
Nausea/vomiting	5/9 (56%)	2/8 (25%)	0.314
Weight loss	0/9 (0%)	0/8 (0%)	>0.999
BRBPR	1/9 (11%)	0/8 (0%)	>0.999
Extraintestinal manifestations			
Head fog/headaches	1/9 (11%)	1/8 (12.5%)	>0.999
Fatigue	5/9 (56%)	2/8 (25%)	0.334
Dermatitis herpitiformis	0/9 (0%)	0/8 (0%)	>0.999
B_12_ deficiency*	0/2 (0%)	1/6 (17%)	>0.999
Vitamin D deficiency	0/0 (0%)	2/4 (50%)	>0.999
Iron deficiency	1/3 (33%)	2/4 (50%)	>0.999
Folate deficiency	0/2 (0%)	0/4 (0%)	>0.999
Transaminase elevation	2/9 (22%)	1/8 (12.5%)	>0.999
TTG IgA			
Mean±SD	1.3±0.23 (n=6)	121.21±80.29 (n=8)	0.003
Median	1.23	105.3	
IgA			
Mean±SD	144.75±41.67	255.5±117.86	0.113
Median	152	233.5	
Upper endoscopy features			
Inflammation	6/9 (67%)	2/6 (33%)	0.153
Mucosal atrophy	1/9 (11%)	2/6 (33%)	0.523
Mucosal ulcers/erosions	1/9 (11%)	2/6 (33%)	0.523
Scalloping	1/9 (11%)	0/6 (0%)	>0.999
Normal duodenum	0/9 (10%)	1/6 (17%)	>0.999
Histological features at diagnosis			
Moderate-to-severe villous blunting	9/9 (100%)	5/6 (83%)	0.4
Increased IELs	2/9 (22%)	4/6 (67%)	0.135
Increased LP cellularity	9/9 (100%)	6/6 (83%)	>0.999
Neutrophilic duodenitis	9/9 (100%)	5/6 (83%)	0.4
Surface erosion/ulceration	5/9 (56%)	5/6 (83%)	0.58

*The p value was calculated by Student’s t-test and analysis of variance method for numerical covariates and Fisher’s exact for categorical covariates where appropriate. Time to symptoms onset is defined as time between the first dose of ICI and the development of symptoms. Combined therapy denotes that patients recieved ipilimumab and a programmed cell death receptor (ligand)-1 (PD-(L)1) inhibitor as standard of care or on an investigational protocol. All laboratory testings listed under ‘extraintestinal manifestations’ were performed between 2 weeks prior to establishing the diagnosis and 1 year after. Vitamin B-12 deficiency was defined as a vitamin B-12 level less than 200 ng/mL. Vitamin D deficiency was defined as a 25-OH vitamin D level less than 20 ng/mL. Iron deficiency was defined as a ferritin level less than 15 ng/mL at any hemoglobin level or a transferrin saturation less than 16%. Folate deficiency was defined as a serum folate concerntration of less than 2 ng/mL. Transaminase elevation were defined as alanine aminotransferase level higher than 33 units/L for males and 29 units/L for females. For patients with a tTG IgA level below the lower limit of the assay (less than 1.2 IU/mL), a level of 1.2 IU/mL was used for statistical calculations. Endoscopic features were all assessed on the first endoscopic evaluation of the patient at presentation. Features of inflammation included erythema, congestion, and granularity. Mucosal atrophy includes features of atrophy and loss of mucosal folds. No pathological change denotes a normal duodenum. All of the boldfaced numbers should be statistically significant and statistical significance is at p value of less than 0.05.

BRBPR, bright red blood per rectum; CTLA-4, cytotoxic T cell associated antigen 4; IEL, intraepithelial lymphocytes; IgA, immunoglobulin A level at diagnosis; LP, lamina propria; tTG IgA, IgA antitissue transglutaminase antibodies at diagnosis.

The diagnosis of ICI-CeD was first established by measurement of tTG IgA (mean: 121.21±80.29 IU/mL). tTG IgA levels were higher in patients with ICI-CeD compared with an in-house cohort of patients with CeD (mean: 82.22±102.48 IU/mL) (table 2 and online supplementary table S1). Patients in the ICI-Duo cohort were defined as having a tTG-IgA within normal limits, or did not have a tTG-IgA drawn and were treated with immunosuppression. IgA levels were normal in all reported patients.

10.1136/jitc-2020-000958.supp1 Supplementary data

A subset of patients who developed ICI-CeD had vitamin D deficiency (n=2/4) and iron deficiency (n=2/4), while one out of six tested had vitamin B_12_ deficiency (table 2). Additionally, the frequency of other irAEs in both patient cohorts was variable (online supplementary table S2) but notably, immune-related hepatitis was more commonly seen in patients with ICI-CeD than in patients with ICI-Duo.

### Endoscopic findings

**Figure 1:**
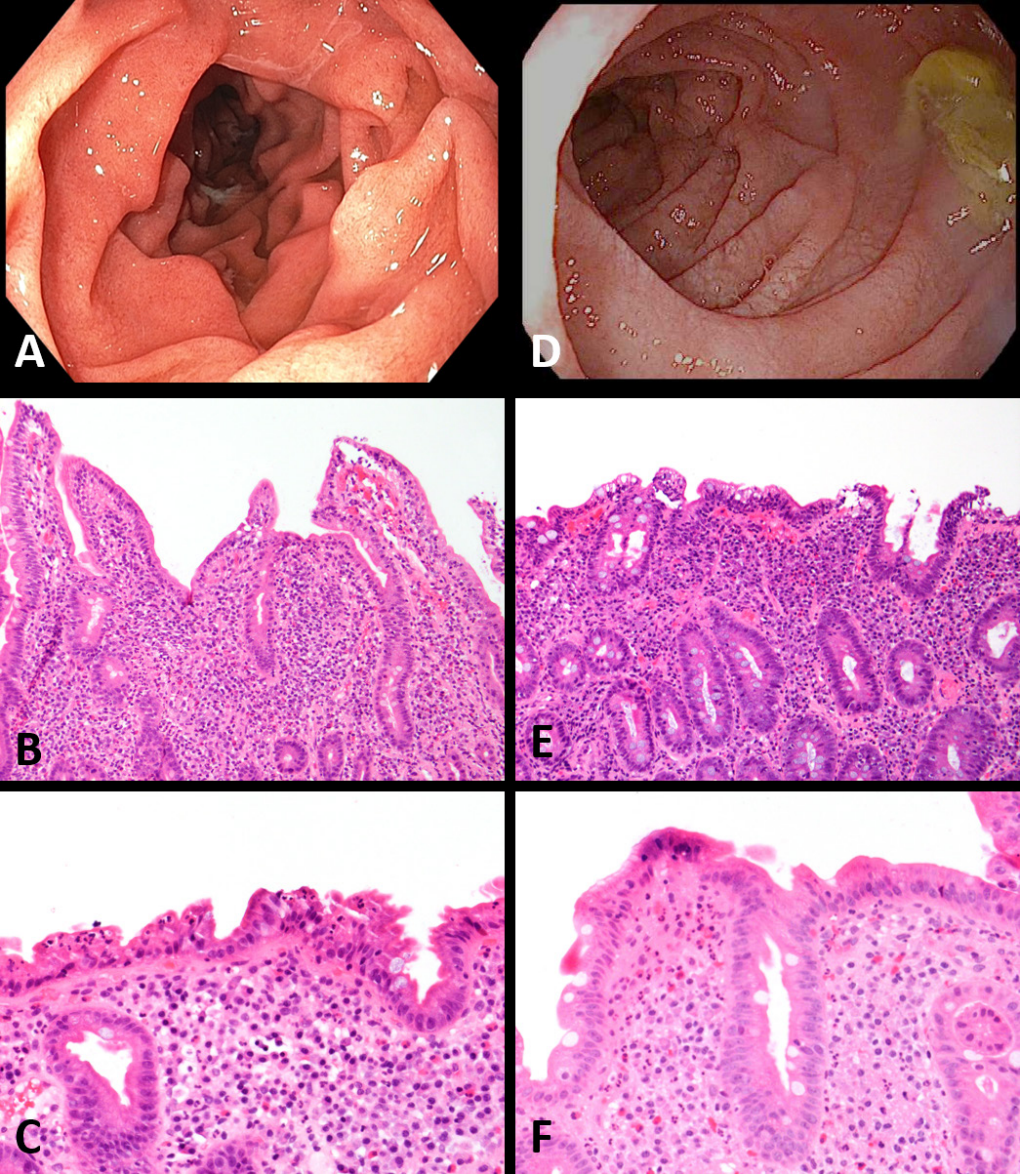
In a patient with immune checkpoint inhibitor (ICI)-associated duodenitis, an endoscopic image of the duodenum reveals diffuse inflammation characterized by congestion, erosions, erythema, and granularity (A). On biopsy, routine H&E showed a markedly active neutrophilic duodenitis with mild-to-moderate villous blunting, marked expansion of the lamina propria, and only mildly increased intraepithelial lymphocytes (B, C). A patient with ICI-celiac disease (CeD) showed endoscopic findings of diffuse inflammation characterized by congestion, erythema, and friability (D). Biopsy of the duodenum showed a mildly active neutrophilic duodenitis with marked villous blunting and increased intraepithelial lymphocytes (E). Intraepithelial lymphocytosis, however, was not present in all ICI-CeD biopsies (F).

Patients presenting with upper GI symptoms (nausea, vomiting, or epigastric pain) without diarrhea underwent an initial upper endoscopy. Patients presenting with diarrhea underwent upper and lower GI endoscopy. Duodenal congestion, nodularity, or erythema were seen on endoscopy in 67% of patients with ICI-Duo, compared with 33% of patients with ICI-CeD (table 2 and figure 1). One patient with ICI-CeD had a grossly normal duodenum on endoscopic examination (table 2). Another patient had a history of asymptomatic CeD and developed a flare after ICI therapy; endoscopic evaluation was not performed in this case, and the patient was managed empirically through dietary modification.

### Histological and immunohistochemical findings

Duodenal biopsies were performed in nine of nine patients with ICI-Duo, and in six of eight patients with ICI-CeD. Histological findings in patients with ICI-Duo universally included moderate-to-severe villous blunting (n=9/9), increased cellularity of the lamina propria (n=9/9), and active neutrophilic duodenitis (n=9/9); surface erosion and ulceration were seen in a slight majority (n=5/9), and intraepithelial lymphocytosis was seen occasionally (n=2/9). Histological findings in the duodenum of patients with ICI-CeD were similar, including moderate-to-severe villous blunting (n=5/6), increased cellularity of the lamina propria (n=6/6), active neutrophilic duodenitis (n=5/6), surface erosion or ulceration (n=5/6), and increased IELs (n=4/6; figure 1 and table 2). The absence of active colitis was confirmed on the colon biopsies from patients who underwent lower GI endoscopy.

**Figure 2:**
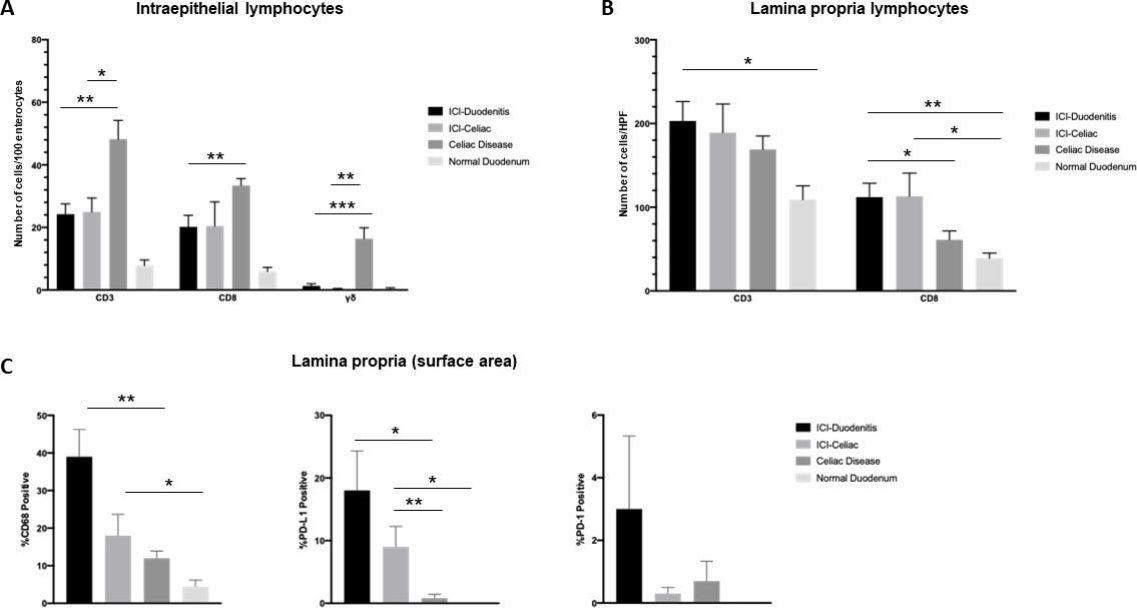
The immunophenotypic characteristics of immune checkpoint inhibitor-associated duodenitis (ICI-Duo) and ICI-celiac disease (CeD) were assessed and quantified in comparison to CeD and normal duodenum. (A) Number of intraepithelial lymphocytes expressed as an average number of CD3, CD8, and δγ positive cells per 100 enterocytes in three well-oriented villi. (B) Number of lamina propria lymphocytes reported as an average number of CD3 and CD8 cells per high power field (HPF; 400×) in three HPFs. (C) Percentage of lamina propria surface area infiltrated by positive cells in the biopsy stained for CD68, programmed death ligand 1 (PD-L1), and programmed death 1 (PD-1). The p value was calculated by the Student t-test and the Welch’s t test for unequal variance, as appropriate. *P<0.05. **P<0.01. ***P<0.001.

**Figure 3:**
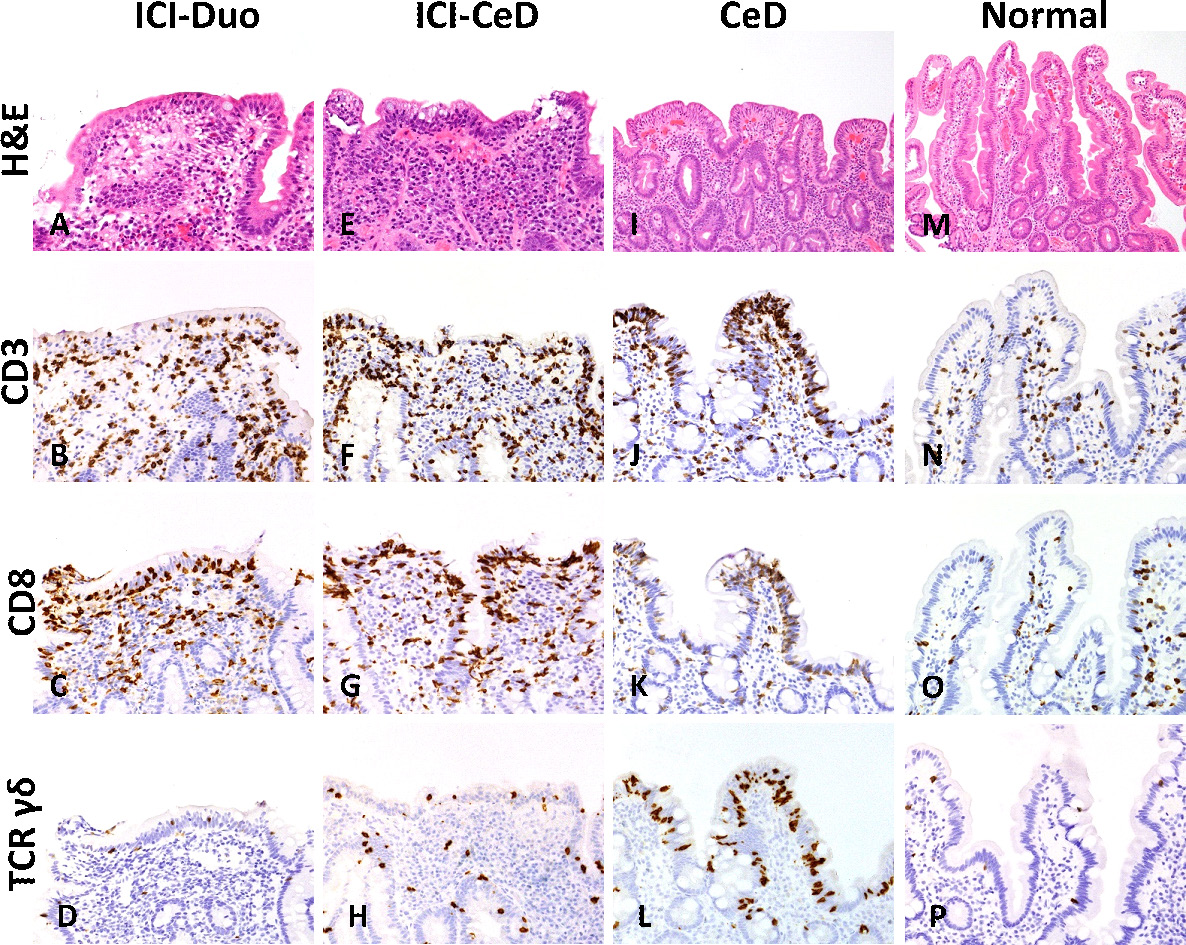
Representative images for (A–D) immune checkpoint inhibitor-associated duodenitis (ICI-Duo), (E–H) ICI-celiac disease (CeD), (I–L) CeD, and (M–P) normal duodenum are shown for routine H&E and immunostains for CD3, CD8, and T-cell receptor (TCR) γδ, respectively. In comparison to normal duodenum, ICI-Duo, ICI-CeD, and CeD show marked villous blunting, with a variably increased intraepithelial CD3+ CD8+ T cells. However, CeD is further characterized by a marked increase in intraepithelial γδ T cells compared with the other groups.

**Figure 4:**
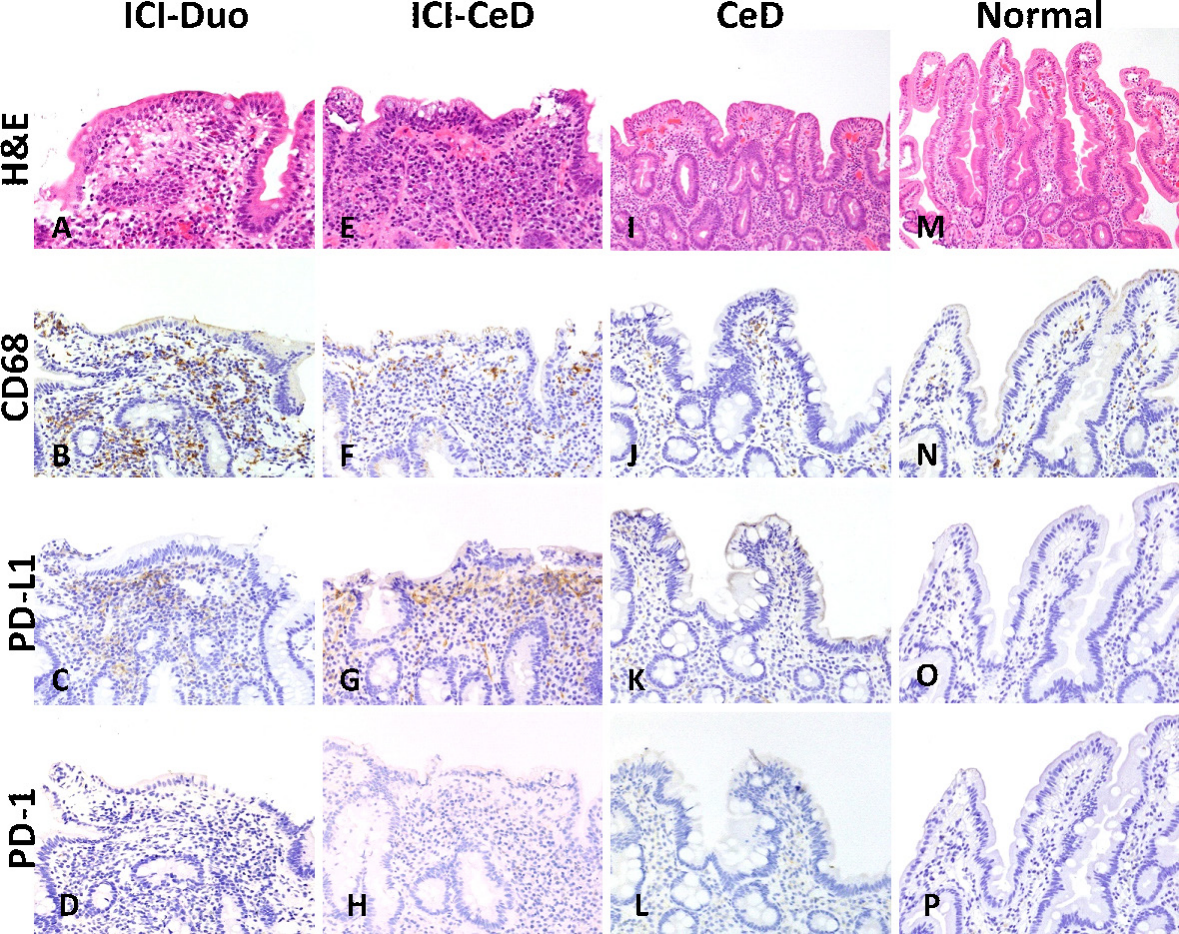
Representative images for (A–D) immune checkpoint inhibitor-associated duodenitis (ICI-Duo), (E–H) ICI-celiac disease (CeD), (I–L) CeD, and (M–P) normal duodenum are shown for routine H&E and immunostains for CD68, programmed death ligand 1 (PD-L1), and programmed death 1 (PD-1), respectively. In comparison to normal duodenum and CeD, patients treated with ICI (ICI-Duo and ICI-CeD) show increased CD68+ macrophages and PD-L1+ cells in the lamina propria. Immunohistochemical evidence of PD-1 expression was uniformly low to absent across all groups.

The immunophenotypic characteristics of ICI-Duo and ICI-CeD were further assessed and quantified in comparison to CeD and normal duodenum (figures 2–4). In ICI-Duo, the average number of IELs per 100 enterocytes was 24 CD3^+^ T cells (± 10 cells); 20 CD8^+^ T cells (± 11 cells); and 1.3 γδ T cells (±2 cells). ICI-CeD had comparable numbers of average IELs per 100 enterocytes, with 25 CD3^+^ T cells (± 11 cells); 20 CD8^+^ T cells (± 19 cells); and 0.2 γδ T cells (±0.6 cells). There was no statistical significance in any of these measures between ICI-Duo and ICI-CeD (p=0.22 to 0.97). However, in comparison to ICI-Duo and ICI-CeD, usual CeD had increased numbers of average IELs per 100 enterocytes, with 48 CD3^+^ T cells (± 19 cells; p=0.0036 and 0.017, respectively); 33 CD8^+^ T cells (± 7 cells; p=0.0058 and 0.067, respectively); and 16 γδ T cells (±11 cells; p=0.00093 and 0.0036, respectively). Normal duodenum had significantly fewer CD3^+^ and CD8^+^ T cells and similar numbers of γδ T cells compared with ICI-Duo and ICI-CeD, with 8 CD3^+^ T cells (±4 cells; p=0.0046 and 0.0093, respectively); 6 CD8^+^ T cells (±3 cells; p=0.013 and 0.12, respectively); and 0.5 γδ T cells (±0.5 cells; p=0.36 and 0.55, respectively; figures 2 and 3).

Quantification of the inflammatory cells of the lamina propria showed that ICI-Duo had an average of 203 CD3^+^ T cells/HPF (±70 cells) and 112 CD8^+^ T cells/HPF (±50 cells), which were similar to ICI-CeD with 189 CD3^+^ T cells/HPF (±84 cells; p=0.72) and 113 CD8^+^ T cells/HPF (±68 cells; p=0.72). Compared with ICI-Duo and ICI-CeD, usual CeD showed similar numbers of CD3^+^ T cells at 169 CD3^+^ T cells/HPF (±51 cells; p=0.24 and 0.57, respectively), but fewer CD8^+^ T cells at 61 CD8^+^ T cells/HPF (±34 cells; p=0.017 and 0.057, respectively). Normal duodenum showed fewer lamina propria T cells compared with ICI-Duo and ICI-CeD, with 109 CD3^+^ T cells/HPF (±37 cells; p=0.016 and 0.08, respectively) and 39 CD8^+^ T cells/HPF (±14 cells; p=0.00085 and 0.04, respectively; figure 2).

Assessment of CD68, PD-L1, and PD-1 was done through an estimate of percent cellularity in the lamina propria (percent lamina propria surface area infiltrated by positive cells; figures 2 and 4). ICI-Duo had an average CD68 percent cellularity of 39% (±22%); PD-L1 percent cellularity of 18% (±19%); and PD-1 percent cellularity of 3% (±7%). ICI-CeD had an average of CD68 percent cellularity of 18% (±14%); PD-L1 percent cellularity of 9% (±8%); and PD-1 percent cellularity of 0.3% (±0.5%), which are not statistically significant compared with ICI-Duo across the board (p=0.27 to 0.054). However, patients with ICI-Duo and ICI-CeD had increased CD68 and PD-L1 populations with similar PD-1 populations compared with CeD, which had an average CD68 percent cellularity of 12% (±6%; p=0.0010 and 0.18, respectively); PD-L1 percent cellularity of 0.8% (±2%; p=0.011 and 0.0068, respectively); and PD-1 percent cellularity of 0.7% (±2%; p=0.40 and 0.59, respectively). Normal duodenum showed smaller CD68, PDL1, and PD1 populations compared with ICI-Duo and ICI-CeD, with an average CD68 percent cellularity of 4.4% (±4%; p=0.06 and 0.035, respectively); PD-L1 percent cellularity of 0% (±0%; p=0.06 and 0.035, respectively); and PD-1 percent cellularity of 0% (±0%; p=0.41 and 0.19, respectively; figure 2).

### Treatment and clinical follow-up

Among patients with ICI-CeD, a gluten-free diet (GFD) was recommended to seven of eight patients (88%). Of these seven patients, five patients were started on a GFD on presentation with clinical remission of disease; in these cases, follow-up endoscopies were not pursued (table 3). The other two patients were administered steroids on presentation, with clarification of diagnosis with subsequent biopsy and tTG-IgA testing. On confirmation of an ICI-CeD diagnosis, these patients were transitioned to GFD alone, resulting in disease remission. In patients with ICI-CeD, tTG-IgA levels down trended (and in one patient decreased to undetectable levels) in response to a GFD (table 3). The single patient who was not trialed on GFD required steroids in addition to infliximab (antitumor necrosis factor (TNF)) for symptom control. A follow-up endoscopy in this patient showed duodenal erosions, and ICI therapy was subsequently terminated.

**Table 3:** Immune checkpoint inhibitor-associated duodenitis (ICI-Duo) and ICI-celiac disease (CeD) treatment and survival

Characteristics	ICI-Duo (n=9)	ICI-CeD (n=8)	P value
Remission-inducing therapy			
GFD	0/9 (0%)	5/8 (62.5%)	0.009
Immunosuppression	9/9 (100%)	1/8 (12.5%)	0.0004
GFD + immunosuppression	0/9 (0%)	2/8 (25%)	0.205
Total duration on steroids if used (weeks)			
Mean±SD	9.50±3.26 (n=8)	32.19±25.36 (n=3)	0.261
Number of steroid tapering attempts			
0	1/9 (11%)	5/8 (62.5%)	0.049
1	5/9 (56%)	1/8 (12.5%)	0.131
2	2/9 (22%)	0/8 (0%)	0.47
>2	1/9 (11%)	2/8 (25%)	0.576
Doses of infliximab to remission (mean)	3 (n=4)	1 (n=1)	N/A
Remission TTG IgA			
Mean±SD	N/A	37.9±57.87 (n=5)	N/A
Median		5.5	
Remission IgA			
Mean±SD	N/A	155.0±57.17 (n=3)	N/A
Median		173	
Need to discontinue immunotherapy, N (%)	6/9 (67)	2/8 (25)	0.153
Number of additional ICI doses received after onset of symptoms			
Mean±SD	0.44±0.73	1.38±1.69	0.151
Median (range)	0 (0–2)	1 (0–5)	
Malignancy restaging (best response)			
Complete response/relapse-free	4/9 (44%)	3/8 (37.5%)	>0.999
Objective response/partial response	1/9 (11%)	2/8 (25%)	0.576
Stable disease	0/9 (0%)	0/8 (0%)	>0.999
Progressive disease/relapsed	4/9 (44%)	3/8 (37.5%)	>0.999
Progression-free survival (months)	Mean: 11±11.29;	Mean: 13.75±16.39;	0.689
	Median: 7; range: 1–29	Median: 7; range: 1–47	
Overall survival (months)	Mean: 22.44±9.70;	Mean: 29.13±15.92	0.306
	Median: 20; range: 13–41	Median: 28; range: 8–52	

The p-value was calculated by Student’s t-test and analysis of variance method for numerical covariates and χ^2^ test or Fisher’s exact for categorical covariates where appropriate. Immunosuppression denotes the use of either corticosteroids or antitumor necrosis factor medications. Remission tissue transglutaminase (tTG) IgA was defined as tTG IgA measured at the time of clinical symptomatic remission or endoscopic remission. Need to discontinue immunotherapy: restricted to complications of ICI-CeD or ICI-Duo and not due to loss of therapeutic effect or disease progression. Malignancy restaging after ICI was determined by the best response per Response Evaluation Criteria In Solid Tumors.

GFD, gluten-free diet; N/A, not applicable.

Only two out of the eight patients who developed ICI-CeD and subsequently received steroids and GFD (n=1) or steroids and infliximab (n=1) were compelled to cease ICI therapy due to the severity of intestinal symptoms. The remaining six patients were able to continue ICI therapy on a GFD. Of these six patients, four patients ultimately discontinued ICI therapy due to disease progression.

In patients who developed ICI-Duo, eight out of nine patients required corticosteroids for symptom control. Four patients needed multiple doses of infliximab to control their disease (median three doses). In six out of nine patients who developed ICI-Duo, the ICI regimen had to be stopped or changed due to the severity of symptoms. Five patients underwent repeat esophagogastroduodenoscopy (EGD) and only two of those patients had endoscopic remission.

Patients who developed ICI-CeD received a higher median number of additional ICI doses administered after development of symptoms compared with patients with ICI-Duo (1 vs 0). One patient with ICI-CeD remained controlled only with GFD and received five additional doses of ICI with good tumor response and no symptom recurrence. Median PFS was 7 months for both ICI-Duo and ICI-CeD. Median OS was 20 months in ICI-Duo compared with 28 months in ICI-CeD (table 3).

## Discussion

Current ICIs induce a wide spectrum of GI toxicities affecting the entire GI tract.^21^ Colitis or enterocolitis are the most frequent GI irAEs, affecting 15%–20% of patients who undergo endoscopic evaluation.^22^ ICI-CeD is less commonly appreciated but has been previously reported.^17 18 20^ Whether ICI-CeD represents de novo disease, or an exacerbation of underlying inflammation as can occur with inflammatory bowel disease, is unclear.^23^

In this study, we provide a clinical and pathological comparison between ICI-CeD and ICI-Duo, finding that the clinical presentation of ICI-CeD and ICI-Duo is very similar, with both pathologies presenting predominantly with abdominal pain and diarrhea. Although ICI-CeD does not appear to have the female predominance that usual CeD has, 25% of patients with ICI-CeD had a family history of CeD.^24^ A personal history of autoimmune disease was not predictive of the development of ICI-CeD, although the size of this study precludes definitive assessment. Similar to findings in published literature, prior exposure to immunotherapy was associated with a higher likelihood of developing ICI-Duo.^16 25^

The histological findings between ICI-Duo and ICI-CeD were similar in the upper GI tract, and in fact closely mimic those of typical CeD with villous blunting, active duodenitis, and patchy increased IELs.^26^ Immunophenotypic characterization showed that ICI-Duo and ICI-CeD have similar quantities of intraepithelial and lamina propria T cells, CD68^+^ macrophages, and PD-(L)1 populations. As expected, typical CeD also showed increased intraepithelial CD3^+^ and CD8^+^ T cells, though with a marked increase in intraepithelial γδ T cells compared with both ICI-Duo and ICI-CeD. Intraepithelial γδ T cells are known to be highly characteristic of conventional CeD and have been proposed as a diagnostic marker.^27–29^ Although the exact role of these lymphocytes in pathogenesis is not completely understood, the difference suggests that ICI-CeD may have a distinct immune etiology, or may represent a distinct phase of CeD that is not typically captured clinically. The pathological similarity between ICI-CeD and ICI-Duo suggest a common immunological mechanism, though likely with distinct antigenic targets.

Patients with ICI toxicities showed a statistically significant increase in the presence of CD68^+^ and PD-L1^+^ macrophages in the lamina propria, compared with both CeD and normal duodenum. These quantitative findings did not appear to correlate with any specific type of ICI therapy, but are consistent with ongoing activation of interferon secreting T cells. The findings of varying populations of immune cells compared with CeD, in addition to the upregulation of PD-L1, also suggest that ICI-CeD and ICI-Duo are mechanistically distinct from conventional CeD. Alternatively, patients who develop ICI-CeD may have a variant of CeD that is controlled by immune checkpoint receptors and evolves to symptomatic disease only in the setting of ICI therapy.

The role of immune checkpoints in CeD pathogenesis has not been specifically studied. Nonetheless, murine models have been used to understand the importance of CTLA-4 and PD-1/PD-L1 in maintaining peripheral tolerance. The impact of PD-1 blockade on CD4 +T cells was studied through adoptively transferring CD4 +T cells harboring an ovalbumin (OVA) restricted T-cell receptor into wild-type mice.^30^ These mice were challenged with OVA peptide to induce tolerance, or with OVA peptide with lipopolysaccharide to induce an immune response while simultaneously blocking PD-1. In these mice, blockade of PD-1 did not prevent establishment of tolerance, nor did it overcome tolerance once it was established. However, in mice that were challenged with OVA and LPS, PD-1 blockade led to enhanced immunity, characterized by increased CD4+ T cell proliferative responses and interleukin 2 and interferon (IFN)-γ production.^30^

In a model in which OVA was expressed as a self-antigen in the small intestine, adoptively transferred naive OVA-specific CD8+ T cells in the setting of a PD-(L)1 blockade led to a breach of self-tolerance. In this setting, OVA-specific CD8+ T cells significantly expanded and produced a proinflammatory signal that led to a histological appearance similar to CeD in humans.^31^ Based on these studies, PD-1/PD-L1 appears to be important in limiting T cell activity in the gut. On the other hand, CTLA-4 is known to be crucial for tolerance induction in the early stages of the immune response as T cells are first presented with antigens by antigen presenting cells (APCs).^32^

We hypothesize that the immune cell activation in the setting of ICIs results in unmasking of gluten sensitivity in genetically susceptible people, leading to expansion of previously self-reactive CD4+ T cells and subsequent CD8+ T cell-induced tissue destruction. Our data suggest a role for both CTLA-4 and PD-1/PD-L1 in the immune regulation of CeD, though the relative importance of each regulatory pathways cannot be determined without a larger cohort, which would likely require a multicenter collaboration.

In the absence of pretreatment tTG-IgA titers, pretreatment histological confirmation, and HLA genotyping, we cannot definitively discern whether the patients in this study had de novo CeD (asymptomatic or subclinical) that progressed to symptomatic CeD in the setting of ICIs.^3^ Additionally, T-cell receptor sequencing to assess for clonal expansion will be crucial in expanding our understanding of ICI-CeD compared with usual CeD and ICI-Duo.

The onset of autoimmune diseases like CeD after therapy with immunomodulatory medications has been previously described.^33^ Some patients with hepatitis C that were treated with IFN-α-based regimens similarly developed CeD, with symptomatic improvement with GFD and cessation of IFN therapy.^34^ Additionally, Gentile *et al* demonstrated the development of ipilimumab-associated CeD in a patient with castration-resistant metastatic prostate cancer, who presented with refractory diarrhea after 3 doses of a CTLA-4 antagonist.^20^ The patient was treated with GFD and immunosuppression with budesonide and prednisone. He was successfully tapered off corticosteroids, and the serum tTG-IgA down trended appropriately. Another group reported the development of ICI-CeD in a patient who received pembrolizumab for locally recurrent melanoma.^17^ The patient developed symptoms within a week of the first infusion of pembrolizumab, and ICI-CeD was confirmed by histology and serum tTG-IgA. ICI therapy was discontinued due to persistence of symptoms, as the patient was unable to adhere to a GFD. Ultimately, the patient’s symptoms resolved with glucocorticoids administered for postural hypotension management.^17^

The responsiveness of ICI-CeD to GFD alone in five out of the eight cases (62%) indicates that gluten is an important antigen driving ICI-CeD, similar to standard CeD, despite the noted phenotypic differences in the immune infiltrate. Intriguingly, the similarities between ICI-CeD and ICI-Duo suggest that duodenitis may result from as of yet unidentified dietary triggers. A management strategy that relies on gluten restriction with monitoring of tTG-IgA and clinical symptoms may be sufficient for patients with ICI-CeD in the absence of gastric or colonic inflammation. Escalation to steroids and anti-TNF therapy can be used as second-line options if GFD fails to control disease activity.

The comparable clinical manifestations, time to symptom onset, and pace of disease progression of the different etiologies of ICI-induced GI toxicities support rigorous diagnostic evaluation in these patients, as clinical differences alone do not seem to be sufficient to determine etiology. The high sensitivity and specificity of tTG-IgA for CeD provide a compelling rationale for testing in all patients with suspected GI toxicities from ICIs early after symptom onset in order to exclude new onset CeD.

We have found that severity of diarrhea assessed by the Common Terminology Criteria for Adverse Events is unable to distinguish the various etiologies of ICI-induced GI toxicities from each other. For this reason, endoscopic biopsies play an integral role in the diagnostic workup of patients with suspected ICI GI toxicities; although most GI toxicities from ICIs occur in the colon, the frequency of upper GI toxicities justifies consideration of esophagogastroduodenocopy alongside a flexible sigmoidoscopy or colonoscopy.^22^ In addition to (entero)colitis and CeD, patients receiving ICI are at risk for pancreatic insufficiency and complications from GI mucosal metastases.^35 36^

The identification of ICI-CeD early in presentation could have substantial clinical implications. A GFD is a reasonable treatment strategy for patients with ICI-CeD (either alone or in combination with immunosuppression), and in many cases may be sufficient as monotherapy. Thus, tailoring therapy can enable patients to limit or avoid systemic immune suppression, and prevent unnecessary premature discontinuation of ICI treatment. Whether identification and treatment of ICI-CeD will have an effect on tumor outcomes is presently unclear, and will require establishing larger, preferably prospective cohorts.
