# Correction: *Immune checkpoint inhibitor-associated celiac disease*

**DOI:** 10.1136/jitc-2020-000958corr1

**Published:** 2020-07-16

Badran YR, Shih A, Leet D, *et al.* Immune checkpoint inhibitor-associated celiac disease. *J Immunother Cancer* 2020;8:e000958. doi: 10.1136/jitc-2020-000958

Since the online publication of this article, the authors have noticed that the middle initial for author Yousef R Badran was missing. This has been corrected.

