# Supplementary material for: Immune checkpoint inhibitor-associated celiac disease
Source: J Immunother Cancer. 2020 Jun 24;8(1):e000958. doi: 10.1136/jitc-2020-000958 (PMC7319774; doi:10.1136/jitc-2020-000958)
Supplement: Supplementary data [file jitc-2020-000958supp001.pdf]

Supplemental Data Tables

Table S1: In-house celiac disease control population characteristics

Characteristics	Celiac Disease
Median age at diagnosis, range (years)	49.5 (40-65)
Sex (F:M)	19:9
TTG IgA at diagnosis<br>Mean +/- SD<br>Median	 82.22 +/- 102.48<br>33.62
IgA at diagnosis<br>Mean +/- SD<br>Median	 246.91 +/- 183.26<br>205.5
Endoscopic features:<br>Inflammation<br>Mucosal atrophy<br>Mucosal ulcers/erosions<br>Scalloping<br>Normal duodenum<br>Other	 6/28 (21.4%)<br>3/28 (21.4%)<br>0/28 (21.4%)<br>3/28 (10.7%)<br>13/28 (46.4%)<br>4/28 (14.3%)
Histologic features at diagnosis<br>Moderate to severe villous blunting<br>Increased IELs<br>Increased LP cellularity<br>Neutrophilic duodenitis<br>Surface erosion/ulceration	 10/10 (100%)<br>10/10 (100%)<br>10/10 (100%)<br>4/10 (40%)<br>0/10 (0%)

Median age at diagnosis: age at endoscopic evaluation that confirmed celiac disease. TTG IgA: IgA anti–tissue transglutaminase antibodies. The first tTG-IgA level measured within a year of endoscopic evaluation was used. For patients with a TTG IgA level below the lower limit of the assay (less than 1.2 IU/mL), a level of 1.2 IU/ml was used for statistical calculations. SD: standard deviation. IgA: Immunoglobulin A level at diagnosis. Histologic features were assessed for ten patients of this cohort. Features of inflammation included erythema, congestion, and granularity. Mucosal atrophy includes features of atrophy and loss of mucosal folds. Other features on endoscopic evaluation: Thickened duodenal folds (n=2), Normal jejunum in a patient who underwent gastrojejunal anastomosis (n=1), duodenal nodularity (n=1). IEL: intraepithelial lymphocytes. LP: lamina propria. No pathologic change denotes a normal duodenum.

Table S2 : Frequency of irAEs in ICI-Duo and ICI-CeD patients

IrAE	ICI-Duodenitis (n=9)	ICI-Celiac Disease (n=8)
Esophagitis	0/9 (0%)	0/8 (0%)
Gastritis	3/9 (33%)	0/8 (0%)
Dermatitis	2/9 (22%)	2/8 (25%)
Hypophysitis	1/9 (11%)	1/8 (12.5%)
Pneumonitis	1/9 (11%)	1/8 (12.5%)
Thyroiditis	3/9 (33%)	1/8 (12.5%)
Meningitis/Encephalitis	0/9 (0%)	0/8 (0%)
Myocarditis/Pericarditis	1/9 (11%)	0/8 (0%)
Hepatitis	1/9 (11%)	3/8 (37.5%)
Arthritis	1/9 (11%)	0/8 (0%)

Uveitis	1/9 (11%)	0/8 (0%)
